# ***International Journal of Molecular Science***: **Best Paper Award 2012**

**DOI:** 10.3390/ijms13044795

**Published:** 2012-04-13

**Authors:** Kathy Lai

**Affiliations:** MDPI AG, Postfach, CH-4005 Basel, Switzerland and MDPI Branch Office, Beijing 101101, China; E-Mail: kathy.lai@mdpi.com; Tel.: +86-10-6280-0830

*International Journal of Molecular Science* is instituting an annual award to recognize the outstanding papers in the area of chemistry, molecular physics and molecular biology published in the *International Journal of Molecular Science*.

We are pleased to announce the first “*International Journal of Molecular Science* Best Paper Award” for 2012. Nominations were made by the Section Editor-in-Chiefs of *International Journal of Molecular Science* from all papers published in 2008. The awards are issued to reviews and articles separately. We are pleased to announce that the following three papers were chosen:

**Article Award:**

First Prize

**Xing Y. Ling, In Y. Phang, David N. Reinhoudt, G. J. Vancso and Jurriaan Huskens**

Supramolecular Layer-by-Layer Assembly of 3D Multicomponent Nanostructures via Multivalent Molecular Recognition

*Int. J. Mol. Sci.* **2008**, *9*(4), 486–497; doi:10.3390/ijms9040486

Available online: http://www.mdpi.com/1422-0067/9/4/486/

Second Prize

**Demberelnyamba Dorjnamjin, Maamaa Ariunaa and Young K. Shim**

Synthesis of Silver Nanoparticles Using Hydroxyl Functionalized Ionic Liquids and Their Antimicrobial Activity

*Int. J. Mol. Sci.* **2008**, *9*(5), 807–820; doi:10.3390/ijms9050807

Available online: http://www.mdpi.com/1422-0067/9/5/807/

**Review Award:**

First Prize

**Vishwanath Patil, Khanh-Quang Tran and Hans Ragnar Giselrød**

Towards Sustainable Production of Biofuels from Microalgae

*Int. J. Mol. Sci.* 2008, *9*(7), 1188–1195; doi:10.3390/ijms9071188

Available online: http://www.mdpi.com/1422-0067/9/7/1188/

We believe that these three exceptional papers are valuable contributions to *International Journal of Molecular Science* and the scientific research field. On behalf of the Prize Awarding Committee and the Editorial Board of *International Journal of Molecular Science*, we would like to congratulate these three teams for their excellent work. In recognition of their accomplishment, Dr. Jurriaan Huskens, Dr. Young K. Shim and Dr. Vishwanath Patil will receive prizes of CHF 1000, CHF 500, and CHF 500, respectively, and the privilege of publishing an additional paper free of charge in Open Access format in the *International Journal of Molecular Science*, after the usual peer-review procedure.

Prize Awarding Committee

*Editor-in-Chief*, Section ‘Bioactives and Nutraceuticals’

**Prof. Dr. Charles Brennan**

Department of Food and Tourism Management, Manchester Metropolitan University, Hollings Faculty, Old Hall Lane, Manchester, M14 6HR, UK

E-Mail: charlesbrennan1@yahoo.co.uk

*Editor-in-Chief*, Section ‘Molecular Pathology’

**Prof. Dr. Kurt A. Jellinger**

Institute of Clinical Neurobiology, Kenyongasse 18, A-1070 Vienna, Austria

E-Mail: kurt.jellinger@univie.ac.at

*Editor-in-Chief*, Section ‘Molecular Recognition’

**Prof. Dr. Ian A. Nicholls**

School of Pure & Applied Natural Sciences, University of Kalmar, SE-391 82 Kalmar, Sweden

E-Mail: ian.nicholls@hik.se

*Editorial Board Member*, Section ‘Molecular Recognition’

**Prof. Dr. Vince Rotello**

Department of Chemistry, University of Massachusetts, 1302-1314 LGRT, 710 North Pleasant Street, Amherst, MA 01003, USA

E-Mail: rotello@chem.umass.edu

